# The association between retinal vascular geometry changes and diabetic retinopathy and their role in prediction of progression – an exploratory study

**DOI:** 10.1186/1471-2415-14-89

**Published:** 2014-07-07

**Authors:** Maged S Habib, Bashir Al-Diri, Andrew Hunter, David HW Steel

**Affiliations:** 1 Sunderland Eye Infirmary – Queen Alexandra Road, Sunderland SR2 9HP, UK; 2 Department of Computing and Informatics, Lincoln University, Brayford Pool, Lincoln LN6 7TS, UK; 3 Institute of Genetic Medicine, University of Newcastle Upon Tyne, Central parkway, Newcastle NE1 3BZ, UK

**Keywords:** Diabetic retinopathy, Retinal vascular geometry, Retinal vascular analysis, Retinal bifurcations

## Abstract

**Background:**

The study describes the relationship of retinal vascular geometry (RVG) to severity of diabetic retinopathy (DR), and its predictive role for subsequent development of proliferative diabetic retinopathy (PDR).

**Methods:**

The research project comprises of two stages. Firstly, a comparative study of diabetic patients with different grades of DR. (No DR: Minimal non-proliferative DR: Severe non-proliferative DR: PDR) (10:10: 12: 19). Analysed RVG features including vascular widths and branching angles were compared between patient cohorts. A preliminary statistical model for determination of the retinopathy grade of patients, using these features, is presented. Secondly, in a longitudinal predictive study, RVG features were analysed for diabetic patients with progressive DR over 7 years. RVG at baseline was examined to determine risk for subsequent PDR development.

**Results:**

In the comparative study, increased DR severity was associated with gradual vascular dilatation (p = 0.000), and widening of the bifurcating angle (p = 0.000) with increase in smaller-child-vessel branching angle (p = 0.027). Type 2 diabetes and increased diabetes duration were associated with increased vascular width (p = <0.05 In the predictive study, at baseline, reduced small-child vascular width (OR = 0.73 (95% CI 0.58-0.92)), was predictive of future progression to PDR.

**Conclusions:**

The study findings suggest that RVG alterations can act as novel markers indicative of progression of DR severity and establishment of PDR. RVG may also have a potential predictive role in determining the risk of future retinopathy progression.

## Background

Diabetic retinopathy (DR) is a leading cause of visual loss in many developed countries [1,2]. Given that eyes with severe non proliferative diabetic retinopathy (NPDR) have a 52% risk of developing PDR within one year and 60% risk of developing high risk PDR within 5 years [3] there is a need in clinical practice for effective risk stratification. Structural and functional changes in the retinal vasculature have been shown in several studies to be closely related to diabetes and DR [4,5]. Population-based cohort studies have described various vascular calibre changes occurring with the development of diabetes [6], or in diabetics with no retinopathy [7], as well as with the development of early retinopathy in type 1 and type 2 diabetics [1,2,8,9], and indeed the progression of DR [7,10-13]. Some of the retinal vascular changes were also found to be significantly associated with progression risk to PDR while controlling for other baseline risk factors [14].

Changes in the retinal vascular calibre represent only a sole parameter of the retinal vascular network and do not convey information regarding the complexity of the retinal vascular branching pattern. According to Murray [15], the optimal vascular architecture achieves the most efficient blood flow transport with minimum energy allowing for maximum vascular diffusion into the surrounding tissues. Alterations in the geometry of the retinal vascular network may thus reflect a state of vascular dysfunction, and might potentially predict disease development [16-19]. Several features representative of the retinal microvasculature geometry have been evaluated in association with various systemic conditions [16-18,20-22]. Being non-dimensional, these features are less likely to be affected by variations in digital image resolution, ocular magnification or differences in refractive errors. In diabetes, recent studies have explored the role of changes in retinal vascular geometry (RVG) as risk markers for incident DR in young type 1 diabetics. Sasongko et al. [23] demonstrated that certain demographic factors such as age and sex as well as diabetes-related factors such as type, and the duration of diabetes were associated with subtle alterations in the RVG parameters including the branching angles. Moreover, other RVG features predicted incident retinopathy after a median of 3.8 years follow up [24].

In this study, we hypothesise that RVG changes are associated with increased severity of DR, and that, alterations in RVG can act as a novel marker in identifying networks at risk of future progression to proliferative retinopathy. We measure RVG at vascular bifurcations and analyse the relationship of these to progression of DR.

## Methods

### Study population

The study was designed in two parts. Firstly a comparative observational study to evaluate the associations of RVG changes in age-matched cohorts of diabetic subjects with different grades of DR. Within this study, the relationships of certain demographic and clinical risk factors with RVG changes in the diabetic population were analyzed. Secondly, a pilot study of longitudinal data to assess the predictive role of RVG changes for subsequent development of proliferative retinopathy, using retrospective data for patients newly presenting with PDR.

The studies were carried in accordance with the declaration of Helsinki (1989) of the World Medical Association. The protocols were approved by the local Sunderland Research Committee (SLREC 1129) and all recruited subjects gave written informed consent.

Study participants were recruited from patients attending the DR clinic at Sunderland Eye Infirmary and the local diabetic retinopathy screening programme centre.

Type I or II diabetic patients aged 25 – 65 years, with different grades of DR and no previous pan retinal laser photocoagulation treatment were invited to participate in the study. The retinopathy grade was classified into; clinically non-detectable, minimal non-proliferative, severe non-proliferative and proliferative diabetic retinopathy.

Exclusion criteria included refractive error beyond +/− 3 dioptres, concurrent ocular pathology including cataract, glaucoma, corneal opacities or any other retinal and optic nerve head pathology. Patients with uncontrolled systemic hypertension with documented blood pressure measurements of more than 140/90 repeatedly recorded within the previous 12 months were also excluded.

For the predictive study, patients who were referred with PDR from the diabetic retinopathy screening service to the hospital between January 2008 – January 2009 were identified. Amongst these cases, patients aged 65 years or less who presented initially to the local diabetic retinopathy screening service with no clinically detectable retinopathy and with at least preceding 6-years screening follow-up period were included (The progressors group). The control group for this study comprised of age matched diabetic patients attending the local screening service with no clinically detectable DR, also with a corresponding preceding 6-year follow-up period and showing no signs of retinopathy progression through this period. (The non-progressors group).

The other inclusion and exclusion criteria were similar to the comparative study. The baseline initial retinal screening images, the final screening images and the screening images captured at the penultimate visits were extracted for each eye and formally graded and analysed according to the study protocol.

For all participants in both studies, one eye per subject was randomly selected for analysis using online randomization programme. The demographic and medical data were extracted from the clinical notes. These included age, sex, type and duration of diabetes, history of hypertension and high cholesterol as well as concurrent history of other diabetic microvascular complications.

### Photographic methods and retinopathy grading

Mydriatic two-field 50° temporal and nasal retinal images were captured for each eye by an experienced ophthalmic medical photographer, according to the study protocol, using a Zeiss FF 450 Plus Fundus camera fitted with a JVC KY-F70B 3CCD digital camera with a resolution of 1360 × 1024 pixels.

All images were assessed for combined field position and image quality according to the NHS diabetic eye screening programme image quality guidelines. Only images with an overall “Good” quality scores were included for the study.

DR was graded for these images by an experienced diabetic retinopathy grader, who was masked to the subjects’ clinical data, according to the developed system for the EURODIAB IDDM complications study adaptation of the modified Airlie House classification [25].

### Retinal vascular geometry analysis

**Figure 1:**
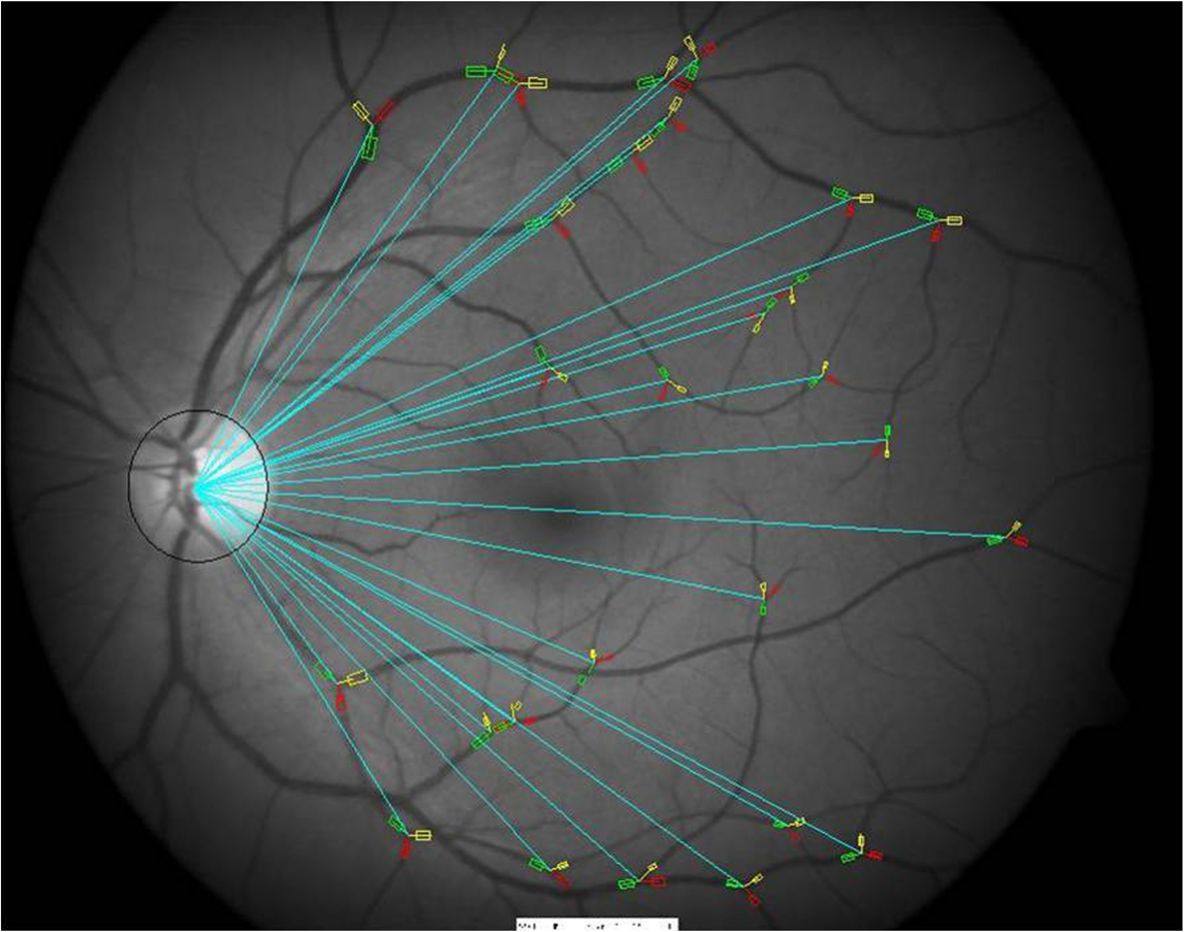
**Distribution of analysed vascular bifurcations.** An example of analysed temporal retinal arteriolar and venular vascular bifurcations of different sizes and orientations evenly spread at variable distances from the optic disc across the fundus image.

**Figure 2:**
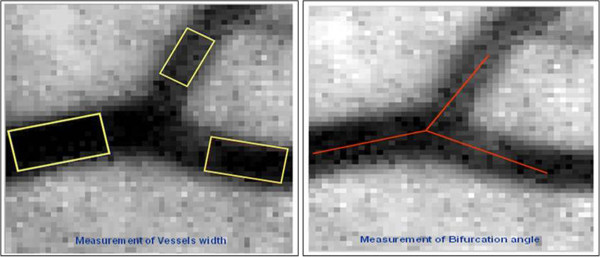
**Semi-manual technique for vascular analysis.** The semi-manual technique: Vascular segment width estimated with aligned rectangles across the vessel longitudinal central axis. The bifurcation angles measured at the intersection point of vascular segments centrelines.

The analysis of the bifurcation geometry was performed utilising a computer assisted semi-manual technique implemented on MATLAB software (MATLAB version 7 0.1 Mathworks Inc. Massachusetts USA), as detailed elsewhere [26].Briefly, for each retinal image, visible arteriolar and venular bifurcations were initially nominated by the observer across the whole image at varying distances from the optic disc, and above and below the horizontal midline. Care was also taken to include a widespread variety of bifurcations of different vascular size (Figure 1). Nominated bifurcations were then marked using the developed tool; the vessel widths were measured by aligning rectangles over each vascular segment centreline and its width adapted to the observed segment edges. The junction angles were estimated by inter-connecting four points; an intersection point and three end-points of the vessels centerlines (Figure 2). The tool presented the junction into a zoomed image allowing the measurements to be obtained at sub-pixel accuracy. For each image an average of 15 bifurcations were measured.

For each analysed vascular bifurcation, the estimated structural and geometrical parameters were: the absolute width measurements of the parent, larger branch and smaller branch vessel segments (*d*_*0*_*, d*_*1*_ and *d*_*2*_ respectively); and the branching angles of the larger and smaller vessel segments, and the overall bifurcation angle (*θ*_*1*,_*θ*_*2*_ and *θ = θ*_*1*_ + *θ*_*2*_ respectively) [16,27].

The precision of the custom-designed technique was compared to other semi-manual techniques used for vascular width detection, namely the “Kick points” [28], “Full width half maximum” [29], “Gregson’s technique” [30], “Single Gaussian algorithm” [31], and the “Two dimensional Gaussian model” techniques [32]. The developed rectangle technique’ results were more precise than other techniques as reflected by lower standard deviation and coefficient of variations values and tighter confidence intervals. Further details were presented in the author’s thesis [33].

A single trained observer, masked to the subjects’ demographic and clinical data, performed all the measurements in the study. The intra-observer repeatability was evaluated on a set of eight images (total of 117 bifurcations) marked three times on separate occasions over a period of one month. The resultant Intra-observer repeatability was assessed using one-way analysis of variance (ANOVA). The average coefficients of variation were 4.2% and 3.8% for the vascular width and angular measurements respectively.

### Statistical analysis

Statistical analysis was performed using Minitab 16 statistical software. Chi Square test *X*^*2*^ was used to detect differences between baseline characteristics. For the comparative study, one-way ANOVA was performed to compare between the subgroups of diabetic cohorts with different grades of diabetic retinopathy. A (*p)* value ≤ 0.05 was considered significant. In cases with significant *(p)* values, the Fisher-protected least significant difference FLSD post-hoc test was used. The results were also sub analysed for arteriolar and venular subgroups.

Multiple linear regression analysis was then used to determine the effect of different demographic and clinical risk factors on the RVG parameters in the diabetic population with or without established retinopathy. Tested features included the continuous variables; age, duration of diabetes and categorical variables; sex, type of diabetes, history of hypertension, and history of hypercholesterolemia. We constructed three models; model 1 included age and sex, model 2 additionally included the diabetes type and history of hypertension, model 3 additionally included diabetes duration and history of hypercholesterolemia.

For the predictive study, one-way ANOVA was used to compare the RVG features between baseline, penultimate and final visits’ images for both the progressors and non-progressors groups, and FLSD post-hoc test was performed for significant relations only where the (*p)* value was ≤ 0.05. Binary logistic regression analysis was used to determine the odd ratios for retinopathy progression based on baseline RVG features measurements of both the progressors and non-progressors groups.

For the diabetic retinopathy grade assessment, a leave-one-out cross validation creating logistic regression models for each patient.

## Results

For the diabetic cross-sectional study, a total of 1518 arteriolar and venular bifurcations of 51 eyes (51 subjects) were analysed. Details of the demographic and clinical baseline characteristics of the included subjects in the different diabetic subgroups are summarised in (Table 1).

**Table 1:** Demographic and clinical baseline characteristics of diabetic subjects

**Demographic and clinical baseline characteristics of diabetic subjects**					
	**No retinopathy group**	**Minimal NPDR group**	**Severe NPDR group**	**PDR group**	**P value**
Subjects	10 Subjects	10 Subjects	12 Subjects	19 Subjects	
(Eyes)	(10 eyes)	(10 eyes)	(12 eyes)	(19 eyes)	
Age					0.92
Mean	55.5	56.6	53	50	
(SD)	(8.9)	(9.5)	(9.0)	(12.5)	
(Range)	(43–65)	(37–65)	(41 – 65)	(26–65)	
Female	60%	50%	42%	58%	0.21
Type II Diabetes	70%	80%	83%	73%	0.70
Duration of DM in months: Mean	118	148	128	211	**<0.05**
(SD)	(65)	(74)	(93)	(124)	
(Range)	(24–144)	(37–240)	(12–300)	(2–456)	
Hypertension	50%	70%	67%	48%	0.74
High Cholesterol	40%	70%	34%	57%	0.16
Associated microvascular complications					0.44
NONE	80%	70%	59%	63%	
IHD, CVA, TIA	10%	20%	0%	5%	
Neuropathy, Foot Ulcer	10%	10%	25%	10%	
Nephropathy	0%	0%	17%	21%	
Smoking	20%	30%	17%	21%	0.8

Duration of DM has a statistically significant correlation with Type of DR, as expected; the other factors do not.

The groups were age-matched with no significant difference in most of their demographic and clinical data. Unsurprisingly, the duration of diabetes was significantly longer in the PDR group as compared to the other subgroups, reflecting an established risk factor for progression of DR.

### Comparative study analysis

For the comparative analysis between diabetic subjects with different DR grades, the distribution of measurements and estimates of the overall analysed retinal geometrical features and parameters in the different subgroups are presented in (Table 2). The results for the arteriolar and venular bifurcations subgroups are presented in (Table 3). The overall significance of CIXthe ANOVA test is shown, along with the significant differences in-between the subgroups as determined by the FLSD test.

**Table 2:** The distribution of geometrical measurements in the diabetic subgroups

**Overall data**					
**Retinal parameter**	**No retinopathy group**	**Mild NPDR group**	**Severe NPDR group**	**PDR group**	**p value**
**Mean ± (STD)**					
	**(242 bifurcations)**	**(310 bifurcations)**	**(372 bifurcations)**	**(594 bifurcations)**	
**Parent Vessel Diameter *d***_***0 ***_**(pixels)**	6.97 ± (1.61)*	8.39 ± (2.37) †	8.56 ± (2.39)†	8.98 ± (2.63)‡	**0.000**
**Large Child Diameter *d***_***1 ***_**(pixels)**	6.17 ± (1.56)*	7.44 ± (2.30)†	7.56 ± (2.34)†	8.01 ± (2.52)‡	**0.000**
**Smaller Child Diameter *d***_***2 ***_**(pixels)**	4.66 ± (1.04)*	5.47 ± (1.53)†	5.51 ± (1.44)†	5.76 ± (1.70)‡	**0.000**
**Bifurcating Angle *θ *** **(Degrees)**	77.04 ± (15.6)*	79.85 ± (17.4)*†	80.02 ± (17.8)†	83.23 ± (18.9)‡	**0.000**
**Branching Angle *θ***_***1 ***_**(Degrees)**	25.14 ± (14.1)	25.12 ± (15.8)	25.82 ± (15.2)	25.05 ± (16.7)	0.941
**Branching Angle *θ***_***2 ***_**(Degrees)**	51.97 ± (19.3)*	55.01 ± (21.2)*	54.84 ± (21.7)*†	58.57 ± (23.7)†	**0.027**
**Junction Exponent** χ	3.33 ± (3.24)*†	3.34 ± (3.17)*†	3.30 ± (3.13)†	3.55 ± (3.32)*	**0.050**

The p-value of ANOVA test is shown with the significant differences between the subgroups as determined by the FLSD test. (Means that do not share a symbol are significantly different).

**Table 3:** The distribution of the arteriolar and venular geometrical measurements in the diabetic subgroups

**Arteriolar data**					
**Retinal parameter**	**No retinopathy group**	**Mild NPDR group**	**Severe NPDR group**	**PDR group**	**p value**
**Mean ± (STD)**	**(117 bifurcations)**	**(136 bifurcations)**	**(139 bifurcations)**	**(231 bifurcations)**	
**Parent Vessel Diameter *d***_***0 ***_**(pixels)**	6.47 ± (1.08)*	7.54 ± (1.68)†	7.58 ± (1.65)†	8.06 ± (1.84)‡	**0.000**
**Large Child Diameter *d***_***1 ***_**(pixels)**	5.78 ± (1.10)*	6.65 ± (1.62)†	6.73 ± (1.66)†	7.23 ± (1.80)‡	**0.000**
**Smaller Child Diameter *d***_***2 ***_**(pixels)**	4.60 ± (0.99)*	5.42 ± (1.37)†	5.43 ± (1.33)†	5.62 ± (1.43)†	**0.000**
**Bifurcating Angle *θ *** **(Degrees)**	76.13 ± (15.8)*	79.14 ± (17.9)*	78.78 ± (18.4)*	84.33 ± (18.6)†	**0.000**
**Branching Angle *θ***_***1 ***_**(Degrees)**	25.77 ± (15.2)	27.32 ± (16.6)	28.33 ± (15.4)	27.44 ± (16.4)	0.560
**Branching Angle *θ***_***2 ***_**(Degrees)**	50.36 ± (20.3)*	51.99 ± (21.3)*	50.45 ± (20.3)*	56.88 ± (22.9)†	**0.014**
**Junction Exponent** χ	3.70 ± (1.27)	3.80 ± (2.00)	3.92 ± (1.85)	4.15 ± (2.25)	0.161
**Venular data**					
**Retinal parameter**	**No retinopathy group**	**Mild NPDR group**	**Severe NPDR group**	**PDR group**	**p value**
**Mean ± (STD)**	**(125 bifurcations)**	**(174 bifurcations)**	**(233 bifurcations)**	**(363 bifurcations)**	
**Parent Vessel Diameter *d***_***0 ***_**(pixels)**	7.42 ± (1.87)*	9.04 ± (2.62)†	9.13 ± (2.56)†‡	9.55 ± (2.88)‡	**0.000**
**Large Child Diameter *d***_***1 ***_**(pixels)**	6.51 ± (1.83)*	8.04 ± (2.55)†‡	8.05 ± (2.54)†	8.50 ± (2.79)‡	**0.000**
**Smaller Child Diameter *d***_***2 ***_**(pixels)**	4.72 ± (1.08)*	5.51 ± (1.64)†	5.55 ± (1.47)†	5.82 ± (1.85)‡	**0.000**
**Bifurcating Angle *θ *** **(Degrees)**	77.68 ± (15.1)	80.40 ± (16.9)	80.75 ± (17.4)	82.53 ± (19.1)	0.062
**Branching Angle *θ***_***1 ***_**(Degrees)**	24.81 ± (12.9)	23.43 ± (14.9)	24.34 ± (15.0)	23.53 ± (16.8)	0.921
**Branching Angle *θ***_***2 ***_**(Degrees)**	53.00 ± (17.9)	57.34 ± (20.9)	57.43 ± (22.1)	59.65 ± (23.7)	0.504
**Junction Exponent** χ	3.02 ± (0.96)	2.99 ± (0.88)	2.94 ± (0.99)	3.18 ± (1.46)	0.07

The p-value of ANOVA test is shown with the significant differences between the subgroups as determined by the FLSD test. (Means that do not share a symbol are significantly different).

There are significant differences in the vessel widths in both arteriolar and venular data, and in the second child branching angle, *θ*_*2*_, in arteriolar data.

The results show that increased DR severity was associated with greater vascular segment width. These relationships are maintained for the arteriolar and venular bifurcations subgroups (p = 0.000).

There is also a widening of the total bifurcation angle (*θ*) with increased severity of DR and development of proliferative retinopathy (p = 0.000). This widening reflected specifically an associated steady increase in the branching angle of the smaller-child vessel segment (*θ*_*2*_) with DR progression (p = 0.027). Such relationships for (*θ*) and (*θ*_*2*_) angles were maintained for the arteriolar bifurcations (p = 0.000, p = 0.014 respectively); however in the venular bifurcations, the same trend of angular widening did not reach a statistically significant level (p = 0.062, p = 0.504 respectively).

### Relationship of RVG with demographic and clinic risk factors

The relationship between the different demographic and clinical risk factors for disease progression and the various RVG features was demonstrated with the multiple regression analysis for the overall measurements and the sub-analysed arteriolar and venular subgroups.

Increased age was associated with significant decreased arteriolar and venular vascular width (p = <0.01) together with reduction in JER (p = <0.01).

Female sex was associated with decreased venular vascular width (p = <0.05) (apart from the diameter of the smaller-child vessel segment) as well as reduction in arteriolar and venular JER (p = <0.05).

Type 2 diabetes was associated with increased arteriolar vascular width (p = <0.05) while the increased duration of diabetes was associated with increased arteriolar and venular vascular width (p = <0.01).

History of hypertension was associated with increased venular vascular width (p = <0.05) (excluding the small-child vessel segment), while history of high cholesterol was associated with increased both the arteriolar bifurcation angle (*θ*) (p = <0.05) and the smaller-child branching angle (*θ*_*2*_) (p = <0.05).

### Model for diabetic retinopathy grade assessment

We conducted a preliminary study to determine if a statistical model can determine the grade of retinopathy given the RVG feature distribution for a particular image (and therefore patient). We used the means of the RVG features to characterize each image. Given the small size of the data set (51 patients), we used the leave-one-out cross validation methodology, constructing logistic regression models to predict the grade of each patient, and training each model using the other patients. We evaluated multinomial models to predict all four grades, but found it more effective to consider two separate types of logistic regression model: model one distinguishes patients without retinopathy from those with retinopathy, and model two distinguishes non-proliferative from proliferative retinopathy.

The RVG parameters vary systematically with distance from the ONH. However, the distribution of bifurcation distances may vary according to the clarity of the images (with a larger proportion further from the ONH for clearer images), and the clarity in turn may be correlated with grade. To ensure that the experiments are controlled for distance from ONH we applied the following methodology. First, we identified the patient with the lowest mean distance ratio from ONH (i.e. distance over ONH diameter). Second, for each of the other patients we progressively discarded the most distant (by ratio) bifurcations until the mean distance ratio was just lower than the “minimum” patient. This procedure ensured that the mean ONH distance ratios for patients were all approximately equal.

Model one achieved 97.6% sensitivity and 90% specificity to determine the presence of retinopathy versus no retinopathy, using two predictors: mean parent diameter and mean deflection angle of the smaller child. Adding other features did not improve the model accuracy. Utilizing the mean parent diameter only reduced specificity to 80%, sensitivity remained at 97.6%, indicating that the mean deflection angle made a small contribution to model performance. Utilizing mean deflection angle alone did not yield a usable model. For this model we utilized all 51 patients, with the mild, severe and proliferative classes merged into a single “retinopathy” class.

Model two achieved 63.2% sensitivity and 72.3% specificity to determine proliferative versus non-proliferative retinopathy. This model used mean parent diameter as the sole predictor; adding the mean deflection angle did not improve performance. For this model we utilized the 41 retinopathy cases, distinguishing the 22 non-proliferatives from 19 proliferatives. If the 10 “no retinopathy” cases are also included in the study the model correctly classifies them as non-proliferative, yielding an overall specificity of 81.3%.

### Predictive study analysis

For the predictive study, five patients aged 50 – 65 years (mean = 57 years) were identified with PDR who fulfilled the inclusion criteria. The non-progressors group included five patients aged 47 – 65 years (mean = 59 years). A total of 121 bifurcations for the progressors and 97 bifurcations for the non-progressors were analysed per screening visit.

The distributions of the geometrical measurements and estimates in the progressors group for the three screening visits are shown in (Table 4) together with the results of the one-way ANOVA test. Absolute vascular width comparisons were excluded from the analysis due to significant difference in the used camera systems over the follow-up period with difference in image resolution that could hamper comparisons made in terms of absolute pixel size. The rest of the geometrical measurements, being non-dimensional, thus unaffected by image resolution, are presented.

**Table 4:** Overall Geometrical features of progressors group

**Progressors overall data**				
**Retinal parameter**	**Baseline visit**	**Penultimate visit**	**Final visit**	**p value**
**Mean ± (STD)**	**(No retinopathy)**	**(No or minimal NPDR)**	**(PDR)**	
**Branching Angle *θ***_***1 ***_**(Degrees)**	26.47 ± (14.9)	28.49 ± (15.3)	26.26 ± (13.9)	0.501
**Branching Angle *θ***_***2 ***_**(Degrees)**	51.98 ± (20.5)	49.98 ± (21.0)	54.07 ± (17.6)	0.334
**Junction Exponent** χ	3.102 ± (1.02)	3.01 ± (0.83)	3.104 ± (1.08)	0.721

Means and Standard deviations for the geometrical features in the progressors group baseline, penultimate and final visits for the overall data. P value for ANOVA test is shown.

There was no statistically significant difference noted in any of the analysed RVG features in the progressive group between the three screening visits, however; there was a noted widening of the vascular bifurcating angle (*θ*) together with a corresponding widening of the smaller-child vessel branching angle (*θ*_*2*_) in the final visit with development of PDR – a trend similar to that observed in the cross-sectional comparative study. Such trend was maintained for the arteriolar and venular subgroup analysis (Table 5).

**Table 5:** Arteriolar and venular geometrical features for progressors group

**Progressors arteriolar data**				
**Retinal parameter**	**Baseline visit**	**Penultimate visit**	**Final visit**	**p value**
**Mean ± (STD)**				
**Branching Angle *θ***_***1 ***_**(Degrees)**	24.94 ± (14.0)	25.77 ± (11.6)	24.71 ± (11.9)	0.924
**Branching Angle *θ***_***2 ***_**(Degrees)**	54.58 ± (22.8)	54.47 ± (22.1)	57.87 ± (18.7)	0.675
**Progressors venular data**				
**Retinal parameter**	**Baseline visit**	**Penultimate visit**	**Final visit**	**p value**
**Mean ± (STD)**				
**Branching Angle *θ***_***1 ***_**(Degrees)**	27.72 ± (15.7)	27.72 ± (15.4)	27.47 ± (15.2)	0.995
**Branching Angle *θ***_***2 ***_**(Degrees)**	49.87 ± (18.4)	47.43 ± (17.7)	51.10 ± (16.2)	0.561

Means and Standard deviations for the geometrical features in the progressors group baseline, penultimate and final visits for the arteriolar and venular data. P value for ANOVA test is shown.

For the non-progressors group, no statistically significant difference was noted for any of the analysed RVG parameters between the three screening visits, and no obvious trend in angular width changes could be observed similar to the progressors group.

The predictive value of RVG features at baseline for determining future progression to PDR was evaluated using the binary logistic regression analysis. The results are shown in (Table 6). Reduced arteriolar and venular smaller-child vessel width (*d*_*2*_) at baseline significantly predicted future progression to PDR (OR = 0.73 (95% CI 0.58-0.92)). The results reached statistical significance in most of the subgroup analysis.

**Table 6:** Results of binary logistic regression analysis

**Binary logistic regression analysis**									
	**Overall**			**Arteriolar**			**Venular**		
**Retinal parameter**	**Coeff**	**OR (95% CI)**	**p value**	**Coeff**	**OR (95% CI)**	**p value**	**Coeff**	**OR (95% CI)**	**p value**
*d*_*0*_	−0.025	0.97 (0.84 - 1.14)	0.742	−0.145	0.86 (0.64 - 1.18)	0.352	−0.021	0.98 (0.81 - 1.18)	0.831
*d*_*1*_	−0.007	0.99 (0.84 - 1.17)	0.93	−0.178	0.84 (0.61 - 1.16)	0.279	0.031	1.03 (0.84 - 1.27)	0.768
*d*_*2*_	**−0.318**	**0.73 (0.58 - 0.92)**	**0.007**	**−0.358**	**0.70 (0.49 - 1.00)**	**0.05**	**−0.301**	**0.74 (0.55 - 1.00)**	**0.05**
*θ*_*1*_	−0.001	1.00 (0.98 - 1.02)	0.844	−0.007	0.99 (0.97 - 1.02)	0.574	0.002	1.00 (0.98 - 1.03)	0.858
*θ*_*2*_	0.011	1.01 (1.00 - 1.02)	0.154	0.014	1.01 (1.00 - 1.03)	0.125	0.005	1.01 (0.98 - 1.03)	0.632
χ	−0.118	0.83 (0.68 - 1.01)	0.069	−0.181	0.83 (0.66 - 1.06)	0.131	−0.099	0.90 (0.54 - 1.53)	0.709

Results of binary logistic regression analysis for the overall data, arteriolar and venular subgroups. The coefficient, odds ratios, 95% confidence intervals, and p values are shown. (Significant results in Bold).

## Discussion

In this study, we evaluated the association of RVG alterations with increased severity of DR in age-matched cohorts of adult type 1 and 2 diabetics with established DR ranging from minimal NPDR to PDR.

The main findings of our comparative study revealed a significant increase in all of the arteriolar and venular vascular segments width measurements with increased severity of DR. This was associated with a larger mean deflection angle of the smaller child vessel at bifurcations. The association of increased vascular calibre with retinopathy severity is consistent with results from previous studies which demonstrated vasodilation in adults with type 2 diabetes with increasing diabetes duration. This was proposed to result from vessel wall smooth muscle cell and pericyte loss with vasomotor dysregulation and loss of structural support leading to the sustained dilatation [4,34,35]. The cause of the apparent change in mean bifurcation angle is not known, but may relate to either a departure from optimality or to adaptation to sustain optimality. These results are consistent with Sasongko et al. showing larger arteriolar branching angles in association with longer diabetes duration [23], but further suggest that this is primarily due to increased deflection of the smaller child vessel.

The results from our patient level statistical model study must be treated with caution, given the small sample size. They are indicative that the distribution of vessel diameter measurements, taken at bifurcations, may be useful in identifying the presence of retinopathy and, to a lesser extent, proliferative retinopathy. The mean deflection angle of the child vessels may also be of some predictive value, although this is less clear.

In the predictive study, we attempted to establish the clinical relevance of the comparative study findings on an individual level. The results showed the same trend, previously demonstrated in the comparative study, of bifurcation angular widening with progression of retinopathy and reaching the maximum with established PDR after an average of 6 years follow up. These changes were rather clearer in the arteriolar vascular system than the venular system, yet did not reach statistical significance, which might be related to the small number of recruited subjects.

The predictive study results have also shown that in adult diabetics with initially no clinically detectable retinopathy, alterations in RVG at baseline in the form of narrower arteriolar and venular small-child vessel segment diameter at a bifurcation and a reduced asymmetry and area ratio values may be correlated with future progression to PDR. These results echo the earlier work by Fanucci et al. who suggested that architectural changes in the arterial bifurcations of human vascular networks in the form of narrow angles and normal area ratios or wide angles and reduced area ratios may predispose to future vascular diseases [36].

Our results also demonstrate the effect of different demographic and clinical risk factors on the RVG features in adult diabetic subjects with various stages of existing retinopathy. Age was associated with reduced arteriolar and venular vascular widths, corresponding to earlier work by Klein et al. on type 1 and type 2 diabetics [7,37]. Longer duration of diabetes was associated with arteriolar and venular dilatation. The increased arteriolar bifurcating angles noted here with high cholesterol levels supports the evolving evidence in previous studies of the role of lipids on the retinal microvasculature [38].

In this study, type 1 and type 2 diabetic patients were included for analysis. Type 2 diabetes was found to be associated only with increased arteriolar vascular width, with no other bearing on the rest of the analysed RVG features. There are no other previous reports that suggested definite comparative difference in retinal vascular analysis between both types of diabetes. In fact, in a recent study evaluating neural and vascular alterations in adolescent type 1 and type 2 diabetic retinae, there was no statistically significant difference in the arteriolar and venular widths, retinal function as measured by multifocal electroretinogram or retinal thickness as detected by the optical coherence tomography noted between both diabetic groups [39].

It is therefore worth noting here that the observed alterations in RVG associated with the various risk factors would have not affected our previous comparative and predictive results, as the study diabetic subgroups were evenly matched in most of their demographic and clinical data (Table 1).

To the best of our knowledge, this is the first study to report on the RVG alterations in adult diabetics with various degrees of DR from mild retinopathy to established proliferative retinopathy. The study provides cross-sectional comparative analysis as well as a pilot predictive analysis for these variations with increased retinopathic severity. Most previous studies have used other semi-manual techniques of retinal vascular measurement that have focused on limited zones surrounding the optic disc [40]. In contradistinction, this study analysed vascular bifurcations of different size and orientation across the entire retina that could arguably be more representative of the retinal vascular network state in normal and disease states, especially with peripheral retinal ischaemia associated with progressive DR. The study’s vascular measurements were performed by a robust and reliable technique and evaluations were conducted by a single observer, masked to the retinopathy grade, to eliminate the inter-observer variability of measurements and reduce sources for bias.

The limitations of the study reside in the fact that, despite our efforts for accurate data collection, it relies on a limited amount of retrospective clinical data. The computer-assisted semi-manual technique was time consuming and labour intensive and thus limited the number of subjects. Future larger-scale studies, utilising reliable and more robust fully automated techniques of vascular analysis, are needed which would allow detailed statistical analysis to be conducted on large samples at the bifurcation-level and the patient-level. The results of the pilot predictive study are also limited by the small number of included patients that fitted the inclusion criteria; however this reflects the rarity of such patients as previously demonstrated in the EURODIAB complication study where only 4% of subjects without diabetic retinopathy at baseline were associated with progression to PDR after follow-up period of 7.3 years [41]. We also note that the expected RVG parameters distribution varies with distance from the ONH, and this distribution may vary between retinopathy grades for reasons other than pathological changes to individual bifurcations, including image clarity. Similarly, measurements such as vessel widths may be affected by media opacity. Although we controlled for the distance from the ONH in our patient level models, the approach does not admit for absolute consistency between patients having different retinal vascular geometry, making it difficult to ensure that the results do not partially reflect some measurement and/or statistical artifacts.

Finally, in our results, it was noted that JER values suffered from over-sensitivity and wide scatter. This over-sensitivity of its calculations to minor variation in vessel width estimations might explain our wide range of measurements and high variability and make it a less than ideal value for investigational studies.

## Conclusion

In conclusion, the study presents novel retinal vascular geometrical markers indicative of progression of DR severity and the establishment of proliferative retinopathy. The results suggest that these markers might allow future quantitative assessment of diabetic microvascular damage and have a potential predictive role in determining the risk of future retinopathy progression. Such findings could help reclassifying patient risk, stratify and alter their follow-up and management plans, and promote early identification of patients at risk of visual loss. The findings warrant further prospective studies to confirm and support these results, aided by the ongoing developments in fully automated vascular analysis software packages.

## Abbreviations

ANOVA: Analysis of variance; DR: Diabetic retinopathy; ETDRS: Early treatment diabetic retinopathy study; FLSD: Fischer-protected least significant difference; JER: Junction exponent ratio; LDR: Length/Distance ratio; NPDR: Non-proliferative diabetic retinopathy; ONH: Optic nerve head; PDR: Proliferative diabetic retinopathy; RVG: Retinal vascular geometry; WESDR: Wisconsin epidemiological study of diabetic retinopathy.

## Competing interests

The authors declare that they have no competing or financial interests. The authors received no external funding support for collecting and analysing the data for this study.

## Authors’ contributions

MH has been involved in designing the study and acquisition of the images datasets, semi-manual marking and analysis of the retinal vascular geometrical features, statistical analysis of data, and drafting the manuscript of this study. BA made extensive contributions in adjusting the custom-designed vascular analysis tool, extracting and interpreting the different vascular features and analysing the data. AH made substantial contribution in developing the computerised software tool, designing the predictive model, analysing the results and drafting and revising the manuscript. DS conceived the study design, provided scientific supervision for the analysis and interpretation of the results, together with extensive contribution to the manuscript draft and critical analysis. All authors read and approved the final manuscript.

## Authors’ information

Mr Maged Habib is a consultant Ophthalmologist and Vitreoretinal Surgeon at Sunderland Eye Infirmary in the North East of England. He has special clinical interest in medical and surgical retinal diseases, specially the management of diabetic eye disease and retinal vascular disorders. He is the lead for virtual diabetic retinopathy surveillance service at South of Tyne Diabetic Eye screening programme. His main research projects explored the role of retinal vascular geometry analysis in the detection and monitoring of diabetic retinopathy as well as other systemic and ocular diseases.

Dr. Bashir Al-Diri is a senior lecturer in School of Computer Science at the University of Lincoln. His main research is concerned with the development of computer vision algorithms to analyse retinal blood vessels for characterization of diabetic retinopathy. Dr. Al-Diri developed a robust fully automated system for retinal vascular segmentation and measurement, which provides a unique combination of good segmentation and superior measurement performance. It is thus uniquely well-suited to act as a base for research into the diagnosis of vascular diseases that cause measurable changes to the geometry of retinal vessels. Dr. Al-Diri research includes computer vision, medical image analysis, automated surveillance, artificial intelligence, speech recognition, language corpus and lexical analysis, and applications of active contour models.

Prof. Andrew Hunter is Pro Vice Chancellor, Head of the College of Science, at the University of Lincoln. He has published over 100 academic papers, including many in international journals. He has also developed several freeware and commercial artificial intelligence software packages. His major research interest is in medical applications of computer vision. He is the Coordinator for the EU Marie-Curie ITN Project “REVAMMAD,” which aims to integrate techniques for retinal vascular modelling, measurement and diagnosis.

Mr David Steel is a Consultant Ophthalmologist at Sunderland Eye Infirmary in the North East of England. He is a vitreoretinal surgeon with clinical interests in all aspects of retinal disease and complex cataract surgery. He is an honorary Senior lecturer at the Institute of Genetic Medicine at University of Newcastle upon Tyne. Mr Steel is also the Consultant lead for the south of the Tyne Diabetic Retinopathy Eye-screening Programme.

## Pre-publication history

The pre-publication history for this paper can be accessed here:

http://www.biomedcentral.com/1471-2415/14/89/prepub
